# Levodopa Reverses Cytokine-Induced Reductions in Striatal Dopamine Release

**DOI:** 10.1093/ijnp/pyu084

**Published:** 2015-01-29

**Authors:** Jennifer C. Felger, Carla R. Hernandez, Andrew H. Miller

**Affiliations:** Department of Psychiatry and Behavioral Sciences, Emory University School of Medicine, Atlanta, GA (Drs Felger, Hernandez, and Miller); The Winship Cancer Institute, Emory University, Atlanta, GA (Drs Felger and Miller)

**Keywords:** dopamine, cytokines, depression, in vivo microdialysis, anhedonia

## Abstract

**Background:**

Studies using neuroimaging and *in vivo* microdialysis in humans and nonhuman primates indicate that inflammatory cytokines such as interferon-alpha reduce dopamine release in the ventral striatum in association with depressive symptoms including anhedonia and psychomotor slowing.

**Methods:**

Herein, we examined whether reduced striatal dopamine release in rhesus monkeys chronically treated with interferon-alpha can be restored by administration of the dopamine precursor levodopa via reverse *in vivo* microdialysis.

**Results:**

Levodopa completely reversed interferon-alpha–induced reductions in striatal dopamine release. No changes were found in the 3,4-dihydroxyphenylacetic acid to dopamine ratio, which increases when unpackaged dopamine is metabolized via monoamine oxidase.

**Conclusions:**

These findings suggest that inflammatory cytokines reduce the availability of dopamine precursors without affecting end-product synthesis or vesicular packaging and/or release and provide the foundation for future studies investigating therapeutic strategies that facilitate availability of dopamine precursors to improve depressive symptoms in patient populations with increased inflammation.

## Introduction

Novel approaches to understanding the neural circuitry of behavior (ie, optogenetics) have revealed that dopamine neurons play a pivotal role in multiple depressive symptoms (Tye et al., 2013). In addition, new methods to probe motivation and reward circuitry in humans have rekindled interest in dopamine in depression (Treadway and Zald, 2011). One pathophysiologic pathway that may influence dopamine in depression is inflammation.

Inflammatory cytokines and other biomarkers of inflammation are reliably elevated in a significant proportion of depressed patients, and peripheral administration of inflammatory cytokines, including interferon (IFN)-alpha, is well known to induce depressive-like behavior in both humans and nonhuman primates (Capuron et al., 2012; Felger and Miller, 2012). Indeed, chronic administration of IFN-alpha to patients with hepatitis C or malignant melanoma produces clinically significant depressive symptoms in up to 50% of treated patients that prominently features reduced motivation and psychomotor slowing (Felger and Miller, 2012), which are difficult to treat with standard antidepressant therapies and are often associated with dopamine depletion (Dunlop and Nemeroff, 2007). Inhibition of inflammatory cytokines such as tumor necrosis factor also has been shown to reduce depressive symptoms, including anhedonia and psychomotor retardation in patients with inflammatory disorders and in depressed patients with increased inflammation (Tyring et al., 2006; Raison et al., 2013).

Further relevant to dopamine and motivation and motor activity, neuroimaging studies in humans have found that administration of IFN-alpha or other stimuli that induce inflammatory cytokines (eg, vaccination or endotoxin) decrease neural activation of the ventral striatum to hedonic reward and alter activity of the substantia nigra, leading to depressive symptoms including anhedonia and psychomotor slowing (Brydon et al., 2008; Eisenberger et al., 2010; Capuron et al., 2012). Furthermore, studies using positron emission tomography have revealed increased uptake and decreased turnover/release of the radiolabeled dopamine precursor, [18F]fluorodopa, in the ventral striatum of patients administered IFN-alpha for hepatitis C, which correlated with IFN-alpha–induced symptoms of depression, including reduced motivation (Capuron et al., 2012). Like levodopa (L-DOPA), [18F]fluorodopa is taken up by dopaminergic neurons and converted into dopamine by DOPA decarboxylase and then stored in synaptic vesicles for release. Increased [18F]fluorodopa uptake indicates that IFN-alpha administration may deplete dopamine precursors, thereby reflexively increasing DOPA decarboxylase activity (which has been observed with neuroleptics and reserpine; (Hadjiconstantinou et al., 1993). Decreased [18F]fluorodopa turnover suggests that newly synthesized dopamine may not be effectively packaged and/or released (Capuron et al., 2012). Our previous work in rhesus monkeys has also demonstrated that chronic IFN-alpha administration decreases striatal dopamine release as measured by reverse in vivo microdialysis stimulation with high potassium or amphetamine (AMPH), which correlated with IFN-alpha–induced decreases in effort-based sucrose consumption, a measure of anhedonic behavior (Felger et al., 2013b). However, whether the effects of IFN-alpha on dopamine release are the result of impaired availability of dopamine precursors or whether cytokines alter dopamine vesicular packaging and/or release mechanisms is currently unknown. Accordingly, in this brief report, we measured turnover and release of dopamine in response to AMPH in the presence or absence of the dopamine precursor, L-DOPA, administered via reverse in vivo microdialysis in rhesus monkeys administered IFN-alpha for 4 weeks compared with untreated control conditions (Felger et al., 2013b).

## Methods

### Animals and IFN-Alpha Treatment

Four rhesus monkeys (*Macaca mulatta*), 2 male (12–14kg) and 2 female (7–9kg), aged 12 to 14 years were individually housed in adjacent cages in same-sex colony rooms. Animals were fed Purina monkey chow twice daily supplemented with fresh fruits and vegetables and maintained on a 7 am-7 pm light-dark cycle. Prior to treatment initiation, animals were habituated to study procedures to minimize stress reactivity. IFN-alpha (rHu-IFN-α-2b, Schering-Plough, Kenilworth, NJ) 20 MIU/m^2^ was subcutaneously administered in equivalent volumes (0.5–1.5mL) between 7 and 10 am 5 days per week for 4 weeks, similar to the treatment schedule of patients receiving IFN-alpha monotherapy for malignant melanoma, which has been previously reported to induce huddling, a depressive-like behavior, and decrease effort-based sucrose consumption in rhesus monkeys (Felger et al., 2007 2013b). See supplementary Materials for additional details regarding the animals and study procedures. All study procedures were a priori approved by the Emory Institutional Animal Care and Use Committee.

### Study Design

**Figure 1.**
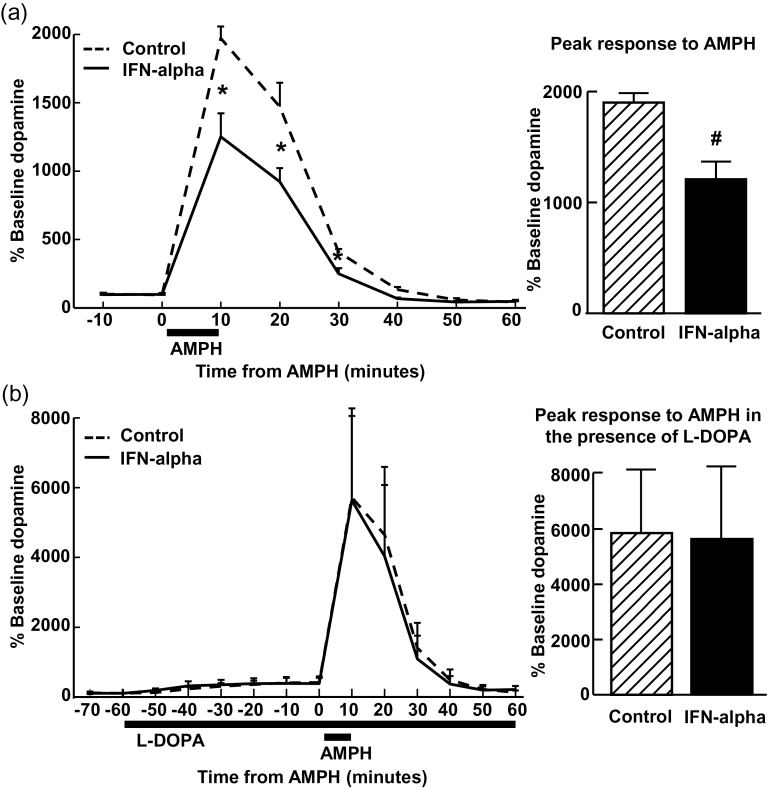
Decreased striatal dopamine release induced by interferon (IFN)-alpha was reversed by levodopa (L-DOPA). IFN-alpha (20 MIU/m^2^ subcutaneously) was administered to 4 rhesus monkeys for 4 weeks, and reverse in vivo microdialysis sampling with amphetamine (AMPH; 100 μM) stimulation was conducted in the presence or absence of the dopamine precursor, L-DOPA (10 μM administered by reverse in vivo microdialysis) and compared with untreated control conditions. IFN-alpha administration significantly decreased extracellular dopamine in response to AMPH stimulation compared with control (a). Administration of L-DOPA produced a similar increase in extracellular dopamine in both IFN-alpha and control conditions and restored IFN-alpha-induced reductions in dopamine release following stimulation with AMPH to control levels (b). Data are presented as mean ± SEM. *P<.05 Tukey’s posthoc test, IFN-alpha compared with control. **#** *P*<.05 paired *t* test, IFN-alpha compared with control.

To assess turnover and release of dopamine in the presence or absence of L-DOPA during IFN-alpha administration, we first tested a range of 4 doses of L-DOPA (1, 10, 100, and 100 μM) previously used in reverse in vivo microdialysis studies in rodents and monkeys in 2 animals to determine the lowest dose that increased extracellular dopamine >2-fold (see supplementary Materials and Figure S3 for details regarding L-DOPA dosing). After collection of data under control conditions, animals were given a recovery period of 3 to 4 weeks prior to initiation of IFN-alpha administration. Animals were assigned to stimulation with AMPH (100 μM) in both the presence and absence of L-DOPA (10 μM) using a counterbalanced design during the last 3 to 4 weeks of IFN-alpha administration, with sampling sessions occurring 10 to 12 days apart (see supplementary Materials and Figure S2). Because of experimental complications during data collection under control conditions in one animal, n=3 for comparison of AMPH stimulation between IFN-alpha and control in the absence of L-DOPA (Figure 1), and n=4 for all other comparisons.

### In Vivo Microdialysis

Subjects were surgically prepared with guide cannulae implanted bilaterally above the caudate nucleus as previously described and in detail in the supplementary Materials (Felger et al., 2013b). The caudate was chosen because of the prominence of motor slowing in IFN-alpha–treated patients and decreased locomotor activity and exploratory behavior in rhesus monkeys observed in previous studies (Felger et al., 2007; Felger and Miller, 2012). Furthermore, the caudate has a similar neurochemical composition as the nucleus accumbens, but its larger size permits a greater likelihood of accurate probe placement. Cannula placement was verified for each animal by MRI (Felger et al., 2013b). Awake subjects underwent reverse in vivo microdialysis sessions with 100 µM AMPH (Sigma, St. Louis, MO) dissolved in artificial cerebrospinal fluid (CSF) for 10- and 60-minute sample collection in the presence or absence of L-DOPA (Sigma), which was infused for 60 minutes prior to AMPH stimulation. Dopamine and 3,4-dihydroxyphenylacetic acid (DOPAC) levels were determined as nanomolar concentrations in dialysate unadjusted for probe recovery (Felger et al., 2013b). The dopamine responses to AMPH were calculated as percent of baseline (extracellular dopamine concentration/mean of 5 baseline samples×100). Samples were analyzed by high-performance liquid chromatography and mass spectroscopic detection (see supplementary Materials for details).

### Statistics

Two-way repeated-measures analysis of variance was used to assess dopamine responses before, during, and after AMPH in each treatment condition (control or IFN-alpha). Sphericity was computed using Mauchly’s W, and if sphericity was violated (Epsilon <0.75), the more conservative Greenhouse-Geisser correction was employed to adjust the degrees of freedom, thus preventing inflation of the F-ratio and reducing the Type I error rate. All tests of significance were 2-tailed with an alpha level of 0.05, and all post hoc analyses were conducted using Tukey’s test or paired *t* test. Statistical analyses were conducted using SPSS (IBM, Armonk, NY) and Sigma Stat (Systat, Jan Jose, CA) software packages.

## Results

Because the dopamine response in the presence of L-DOPA was unknown but anticipated to be high, particularly under control conditions, the dose of AMPH used in our previous study was reduced from 250 to 100 μM (Felger et al., 2013b). In the untreated control condition, the 100-μM dose of AMPH produced robust increases in dopamine from baseline (Figure 1a), consistent with previous studies (Gerhardt et al., 2002; Felger et al., 2013b). As previously reported, in the absence of L-DOPA, IFN-alpha significantly decreased dopamine release to AMPH stimulation compared with control, with a significant effect of treatment (F[1,2]=724.4, *P*=.001) and a significant treatment ×time interaction (F[1.1,2.2]=377.30, *P*=.02) (Felger et al., 2013b) (Figure 1a). Administration of L-DOPA (10 μM) completely reversed IFN-alpha effects on dopamine release following stimulation with AMPH, which produced similar increases in extracellular dopamine in both IFN-alpha and control conditions, with no effect of treatment (F[1,3]=0.16, *P*=.72) or treatment ×time interaction (F[1.2,3.7]=0.33, *P*=.61) (Figure 1b).

**Figure 2.**
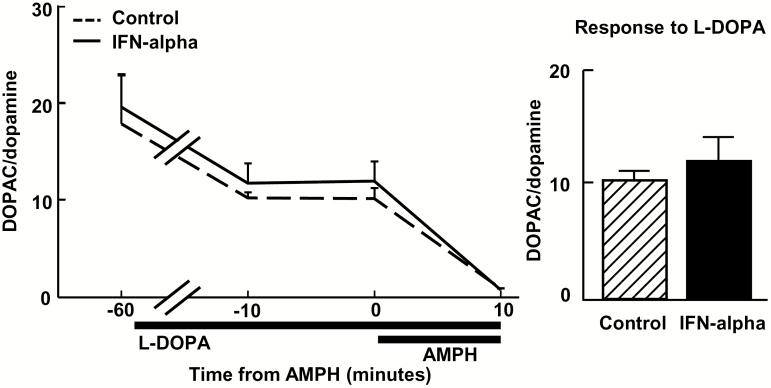
Administration of levodopa (L-DOPA) during interferon (IFN)-alpha administration is not associated with increased 3,4-dihydroxyphenylacetic acid (DOPAC) to dopamine ratio. IFN-alpha (20 MIU/m^2^ subcutaneously) was administered to 4 rhesus monkeys for 4 weeks, and reverse in vivo microdialysis sampling with amphetamine stimulation (AMPH; 100 μM) was conducted in the presence of the dopamine precursor, L-DOPA (10 μM; administered by reverse in vivo microdialysis), and DOPAC to dopamine ratio (DOPAC/dopamine) was compared with control conditions before and after L-DOPA followed by AMPH administration. DOPAC/dopamine did not differ between control and IFN-alpha conditions either before or after administration of L-DOPA and AMPH, indicating that dopamine packaging and release mechanisms were intact following IFN-alpha administration. Data are presented as mean ± SEM.

In regard to potential effects of IFN-alpha on vesicular packaging and release, newly synthesized dopamine that is not packaged into synaptic vesicles is broken down into DOPAC in the extravesicular space by monoamine oxidase (Caudle et al., 2007). If dopamine packaging and release mechanisms were affected by cytokine exposure, the DOPAC to dopamine ratio (DOPAC/dopamine) would be expected to be elevated during IFN-alpha administration. However, DOPAC/dopamine, which decreased following L-DOPA administration, did not differ between control and IFN-alpha conditions either before or after L-DOPA and AMPH administration, with no effect of treatment (F[1,3]=0.20, *P*=.69) or treatment ×time interaction (F[1.1,3.2]=0.09, *P*=.80) (Figure 2).

## Discussion

Reversal of the IFN-alpha–induced decrease in dopamine release by L-DOPA in the absence of an increase in DOPAC/dopamine provides evidence that cytokines affect dopamine precursors without affecting end-product synthesis or vesicular packaging or release mechanisms. These findings are consistent with observations of decreased CSF concentrations of dopamine metabolites (DOPAC and/or homovanillic acid) in both humans and monkeys exposed to chronic IFN-alpha (Felger et al., 2007; Felger and Miller, 2012).

Administration of IFN-alpha induces a number of other inflammatory cytokines in the periphery and central nervous system (CNS) that are elevated in depression, including interleukin-6 and tumor necrosis factor (Raison et al., 2009). IFN-alpha and other cytokines can then activate inflammatory mediators in the CNS that affect neurotransmitter systems, such as neuroactive metabolites of the kynurenine pathway and nitric oxid (NO) (Kitagami et al., 2003; O’Connor et al., 2009). One mechanism by which IFN-alpha and other inflammatory cytokines may reduce dopamine precursors in the CNS is by effects on tetrahydrobiopterin (BH4), an enzyme cofactor required for conversion of phenylalanine to tyrosine by phenylalanine hydroxylase and tyrosine to L-DOPA by tyrosine hydroxylase (Neurauter et al., 2008). BH4 is also a cofactor for NO synthases (NOS), and cytokine-induced increases in inducible NOS can usurp available BH4, resulting in NOS uncoupling and the generation of reactive oxygen species rather than NO (see Felger and Miller, 2012 for discussion). Inflammation and inducible NOS-related decreases in BH4 can further increase oxidative stress and contribute to oxidative reduction of BH4 itself (which is highly redox-sensitive), leaving even less BH4 available for dopamine synthesis. Indeed, intramuscular injection of IFN-alpha to rats has been shown to decrease CNS concentrations of both dopamine and BH4 through stimulation of NO, and treatment with a NOS inhibitor reversed IFN-alpha’s inhibitory effects on BH4 and dopamine in the brain (Kitagami et al., 2003).

We and others have previously reported evidence of reduced BH4 activity in IFN-alpha–treated patients (Zoller et al., 2012; Felger et al., 2013a). For example, IFN-alpha administration is associated with increased peripheral blood phenylanine to tyrosine ratio, which in turn correlated with decreased CSF dopamine and homovanillic acid (Zoller et al., 2012; Felger et al., 2013a). Increased CSF interleukin-6 was also correlated with decreased BH4 in CSF of IFN-alpha–treated patients (Felger et al., 2013a). Of note, the phenylalanine to tyrosine ratio significantly correlated with IFN-alpha–induced depressive symptoms (Felger et al., 2013a). Moreover, dietary depletion of dopamine precursors including phenylalanine and tyrosine has been found to decrease neural activation of the ventral striatum to hedonic reward (Bjork et al., 2014), similar to that observed following administration of IFN-alpha or endotoxin (Eisenberger et al., 2010; Capuron et al., 2012).

Together, these findings suggest that pharmacologic strategies that boost key components of dopamine synthesis may be efficacious, whereas dopamine reuptake inhibitors may be less effective, in treating depressive symptoms in patients with increased inflammation. Indeed, dopamine reuptake inhibitors have demonstrated limited efficacy in the treatment of depressive symptoms such as fatigue in cancer patients (Moraska et al., 2010; Bruera et al., 2013), as well as those with other medical disorders associated with inflammation (see Felger and Miller, 2012 for discussion). Several pharmacological strategies exist to increase BH4 (see Felger and Miller, 2012, for discussion), including administration of BH4 itself, which may bolster dopamine synthesis. Additionally, folic acid, l-methylfolate, or *S*-adenosyl-methionine all have a role in the synthesis and/or regeneration of BH4. Even antioxidants, such as citric acid, have been shown to protect BH4 from oxidation and may be relevant to treatment of BH4 and dopamine deficiency (Heller et al., 1999). Finally, restoration of dopamine directly through administration of L-DOPA/carbidopa may reverse depressive symptoms in patients with increased inflammation and low dopamine, as has been observed in patients with Parkinson’s disease (Brodell et al., 2012).

There are several limitations of this brief report that should be addressed, the first being the small samples size. However, 3 to 5 animals per group is common in nonhuman primate studies employing in vivo microdialysis techniques, and as with previous studies, a within-subject design was used to reduce effects of inter-subject variability and increase statistical power (Felger et al., 2013b). An additional limitation is that this study was designed to examine only whether the dopamine precursor L-DOPA could restore cytokine-induced reductions in dopamine release, which were previously found to correlate with decreases in effort-based sucrose consumption, a measure of anhedonia (Felger et al., 2013b). Future studies will be necessary to investigate whether restoration of dopamine release by agents that increase dopamine synthesis can reverse cytokine-related behavioral symptoms in humans or nonhuman primates.

In conclusion, the findings of this brief report indicate that cytokine-induced reductions in striatal dopamine release can be restored by administration of L-DOPA. These findings directly extend our previous findings of decreased dopamine release and decreased CSF dopamine metabolites in rhesus monkeys administered chronic IFN-alpha and complement neuroimaging studies in humans indicating that administration of cytokines or cytokine-inducers alter ventral striatal activation and dopamine metabolism to produce depressive symptoms of anhedonia and psychomotor slowing. Multiple pharmacological treatment strategies exist that may facilitate the availability of dopamine precursors, such as compounds that restore BH4 activity, and future studies are needed determine the potential for these therapies to improve depressive symptoms in patients with increased inflammation.

## Supplementary Material

For supplementary material accompanying this paper, visit http://www.ijnp.oxfordjournals.org/

## Statement of Interest

None.

## References

[CIT0001] Bjork JM Grant SJ Chen G Hommer DW (2014) Dietary tyrosine/phenylalanine depletion effects on behavioral and brain signatures of human motivational processing. Neuropsychopharmacology 39:595–604. 23995581 10.1038/npp.2013.232 PMC3895237

[CIT0002] Brodell DW Stanford NT Jacobson CE Schmidt P Okun MS (2012) Carbidopa/levodopa dose elevation and safety concerns in Parkinson’s patients: a cross-sectional and cohort design. BMJ Open 2. 10.1136/bmjopen-2012-001971 PMC3533055 23233700

[CIT0003] Bruera E Yennurajalingam S Palmer JL Perez-Cruz PE Frisbee-Hume S Allo JA Williams JL Cohen MZ (2013) Methylphenidate and/or a nursing telephone intervention for fatigue in patients with advanced cancer: a randomized, placebo-controlled, phase II trial. J Clin Oncol 31:2421–2427. 23690414 10.1200/JCO.2012.45.3696 PMC3691358

[CIT0004] Brydon L Harrison NA Walker C Steptoe A Critchley HD (2008) Peripheral inflammation is associated with altered substantia nigra activity and psychomotor slowing in humans. Biol Psychiatry 63:1022–1029. 18242584 10.1016/j.biopsych.2007.12.007 PMC2885493

[CIT0005] Capuron L Pagnoni G Drake DF Woolwine BJ Spivey JR Crowe RJ Votaw JR Goodman MM Miller AH (2012) Dopaminergic mechanisms of reduced basal ganglia responses to hedonic reward during interferon alfa administration. Arch Gen Psychiatry 69:1044–1053. 23026954 10.1001/archgenpsychiatry.2011.2094 PMC3640298

[CIT0006] Caudle WM Richardson JR Wang MZ Taylor TN Guillot TS McCormack AL Colebrooke RE Di Monte DA Emson PC Miller GW (2007) Reduced vesicular storage of dopamine causes progressive nigrostriatal neurodegeneration. J Neurosci 27:8138–8148. 17652604 10.1523/JNEUROSCI.0319-07.2007 PMC6672727

[CIT0007] Dunlop BW Nemeroff CB (2007) The role of dopamine in the pathophysiology of depression. Arch Gen Psychiatry 64:327–337. 17339521 10.1001/archpsyc.64.3.327

[CIT0008] Eisenberger NI Berkman ET Inagaki TK Rameson LT Mashal NM Irwin MR (2010) Inflammation-induced anhedonia: endotoxin reduces ventral striatum responses to reward. Biol Psychiatry 68:748–754. 20719303 10.1016/j.biopsych.2010.06.010 PMC3025604

[CIT0009] Felger JC Miller AH (2012) Cytokine effects on the basal ganglia and dopamine function: the subcortical source of inflammatory malaise. Front Neuroendocrinol 33:315–327. 23000204 10.1016/j.yfrne.2012.09.003 PMC3484236

[CIT0010] Felger JC Alagbe O Hu F Mook D Freeman AA Sanchez MM Kalin NH Ratti E Nemeroff CB Miller AH (2007) Effects of interferon-alpha on rhesus monkeys: a nonhuman primate model of cytokine-induced depression. Biol Psychiatry 62:1324–1333. 17678633 10.1016/j.biopsych.2007.05.026 PMC2149847

[CIT0011] Felger JC Li L Marvar PJ Woolwine BJ Harrison DG Raison CL Miller AH (2013a) Tyrosine metabolism during interferon-alpha administration: association with fatigue and CSF dopamine concentrations. Brain Behav Immun 31:153–160. 23072726 10.1016/j.bbi.2012.10.010 PMC3578984

[CIT0012] Felger JC Mun J Kimmel HL Nye JA Drake DF Hernandez CR Freeman AA Rye DB Goodman MM Howell LL Miller AH (2013b) Chronic interferon-alpha decreases dopamine 2 receptor binding and striatal dopamine release in association with anhedonia-like behavior in nonhuman primates. Neuropsychopharmacology 38:2179–2187. 23657438 10.1038/npp.2013.115 PMC3773667

[CIT0013] Gerhardt GA Cass WA Yi A Zhang Z Gash DM (2002) Changes in somatodendritic but not terminal dopamine regulation in aged rhesus monkeys. J Neurochem 80:168–177. 11796755 10.1046/j.0022-3042.2001.00684.x

[CIT0014] Hadjiconstantinou M Wemlinger TA Sylvia CP Hubble JP Neff NH (1993) Aromatic L-amino acid decarboxylase activity of mouse striatum is modulated via dopamine receptors. J Neurochem 60:2175–2180. 8492125 10.1111/j.1471-4159.1993.tb03503.x

[CIT0015] Heller R Munscher-Paulig F Grabner R Till U (1999) L-Ascorbic acid potentiates nitric oxide synthesis in endothelial cells. J Biol Chem 274:8254–8260. 10075731 10.1074/jbc.274.12.8254

[CIT0016] Kitagami T Yamada K Miura H Hashimoto R Nabeshima T Ohta T (2003) Mechanism of systemically injected interferon-alpha impeding monoamine biosynthesis in rats: role of nitric oxide as a signal crossing the blood-brain barrier. Brain Res 978:104–114. 12834904 10.1016/s0006-8993(03)02776-8

[CIT0017] Moraska AR Sood A Dakhil SR Sloan JA Barton D Atherton PJ Suh JJ Griffin PC Johnson DB Ali A Silberstein PT Duane SF Loprinzi CL (2010) Phase III, randomized, double-blind, placebo-controlled study of long-acting methylphenidate for cancer-related fatigue: North Central Cancer Treatment Group NCCTG-N05C7 trial. J Clin Oncol 28:3673–3679. 20625123 10.1200/JCO.2010.28.1444 PMC2917307

[CIT0018] Neurauter G Schrocksnadel K Scholl-Burgi S Sperner-Unterweger B Schubert C Ledochowski M Fuchs D (2008) Chronic immune stimulation correlates with reduced phenylalanine turnover. Curr Drug Metab 9:622–627. 18781914 10.2174/138920008785821738

[CIT0019] O’Connor JC Andre C Wang Y Lawson MA Szegedi SS Lestage J Castanon N Kelley KW Dantzer R (2009) Interferon-gamma and tumor necrosis factor-alpha mediate the upregulation of indoleamine 2,3-dioxygenase and the induction of depressive-like behavior in mice in response to bacillus Calmette-Guerin. J Neurosci 29:4200–4209. 19339614 10.1523/JNEUROSCI.5032-08.2009 PMC2835569

[CIT0020] Raison CL Borisov AS Majer M Drake DF Pagnoni G Woolwine BJ Vogt GJ Massung B Miller AH (2009) Activation of central nervous system inflammatory pathways by interferon-alpha: relationship to monoamines and depression. Biol Psychiatry 65:296–303. 18801471 10.1016/j.biopsych.2008.08.010 PMC2655138

[CIT0021] Raison CL Rutherford RE Woolwine BJ Shuo C Schettler P Drake DF Haroon E Miller AH (2013) A randomized controlled trial of the tumor necrosis factor antagonist infliximab for treatment-resistant depression: the role of baseline inflammatory biomarkers. JAMA Psychiatry 70:31–41. 22945416 10.1001/2013.jamapsychiatry.4 PMC4015348

[CIT0022] Treadway MT Zald DH (2011) Reconsidering anhedonia in depression: lessons from translational neuroscience. Neurosci Biobehav Rev 35:537–555. 20603146 10.1016/j.neubiorev.2010.06.006 PMC3005986

[CIT0023] Tye KM Mirzabekov JJ Warden MR Ferenczi EA Tsai HC Finkelstein J Kim SY Adhikari A Thompson KR Andalman AS Gunaydin LA Witten IB Deisseroth K (2013) Dopamine neurons modulate neural encoding and expression of depression-related behaviour. Nature 493:537–541. 23235822 10.1038/nature11740 PMC4160519

[CIT0024] Tyring S Gottlieb A Papp K Gordon K Leonardi C Wang A Lalla D Woolley M Jahreis A Zitnik R Cella D Krishnan R (2006) Etanercept and clinical outcomes, fatigue, and depression in psoriasis: double-blind placebo-controlled randomised phase III trial. Lancet 367:29–35. 16399150 10.1016/S0140-6736(05)67763-X

[CIT0025] Zoller H Schloegl A Schroecksnadel S Vogel W Fuchs D (2012) Interferon-alpha therapy in patients with hepatitis C virus infection increases plasma phenylalanine and the phenylalanine to tyrosine ratio. J Interferon Cytokine Res 32:216–220. 22191466 10.1089/jir.2011.0093

